# Measured treatment differences between sexes and genders: A scoping review

**DOI:** 10.1371/journal.pone.0356176

**Published:** 2026-09-16

**Authors:** Miriam Veenhuizen, Andrew O’Malley

**Affiliations:** 1 University of St Andrews, St Andrews, United Kingdom; 2 University of Keele, Newcastle-under-Lyme, United Kingdom; Johns Hopkins University School of Medicine, UNITED STATES OF AMERICA

## Abstract

**Background:**

Sex and gender-based inequality is found throughout medicine. Although there has been significant research in gender disparities affecting healthcare professionals, there is a lack of research into gender differences in the treatment of patients. Despite recognition of systemic issues, recent empirical evidence on how gender influences actual treatment decisions remains fragmented across specialties. A preliminary search of MEDLINE, the Cochrane Database of Systematic Reviews and JBI (Joanna Briggs institute) Evidence Synthesis was conducted and no current or underway systematic reviews or scoping reviews on the topic were identified.

**Methods:**

Studies in English published between 2018 and 2023 were included. Only research which studied measurable differences in the care received by patients of different sexes or genders was included. Both authors independently screened the literature against the a priori defined eligibility criteria. A thematic synthesis approach was used and results summarised qualitatively.

**Results:**

Forty-one papers were included in this review. Of the thirty-eight retrospective case series, thirty-three found a significant difference between the treatment of male and female patients. Of the twenty-nine case series using a multivariate analysis, twenty-five studies found a significant difference in treatment between male and female patients.

**Conclusions:**

The results of this review indicate that men and women are frequently treated differently by healthcare systems. During 2018–2023, five hundred and fifty-one papers were published which studied gender-based differences in opportunities given to healthcare professionals, but only forty-one studying the treatment given to patients. This highlights an area of unmet research need.

## 1. Introduction

Sex inequities are evident throughout medicine. Women remain underrepresented in senior academic roles [1], conference presentations [2], and surgical careers where male-dominated culture and structural barriers persist [3]. Harassment and discrimination during training further entrench disadvantage, with one study reporting that 87% of women in surgery experienced sex-based discrimination in medical school [4]. These inequities extend into pay gaps [5], differential allocation of healthcare resources [6], and even patient interactions, where staff report gender- and race-based abuse [7,8]. Policy initiatives, such as commitments from the Medical Schools Council and General Medical Council [9,10], signal recognition of the problem but persistent disparities remain.

Similar structural inequities also exist from a patient perspective. The field of women’s health has developed considerably since the women’s health movement of the 1960s and 1970s, which centred on reproductive rights and the exclusion of women from clinical research [11]. While the term sex refers to a biological difference between females and males, gender refers to societal views and norms differentiating women from men [12]. Over time, the scope has broadened to encompass sex- and gender-based differences across all populations, though significant challenges remain [11]. Females are still underrepresented in clinical research trials; this results in clinical guidelines being based on data skewed towards male patients [13]. However, some feel that knowledge of sex differences will not result in a reduction in gender discrimination in healthcare [12].

The term administrative sex refers to the classification of a person’s sex on official documentation such as medical records [14]. Current clinical databases have been criticised for failing to allow for recording of a patient’s gender, or for intersex patients [14]. The lack of information in medical databases can make research into sex and gender inequality challenging [14].

Within healthcare, sex plays a role in the diagnosis and management of patients [13]. Research into this phenomenon began in the 1970s, with psychiatric and gynaecologic practise coming under scrutiny [15]. In the 1990s, the concept of the Yentl Syndrome was published, describing poorer outcomes for women following myocardial infarction and differences in clinical presentation [16]. This work helped to catalyse the field of gender medicine, which aims to research sex differences in disease and reduce gender-based management disparities [13,17].

Cardiovascular medicine illustrates many of these ongoing disparities. Females remain substantially underrepresented in clinical trials of cardiovascular drugs, acute coronary syndromes, heart failure and interventional procedures [18]. Similar gaps exist in authorship and leadership, with under-representation of women as investigators being associated with lower recruitment of women as participants [18]. Despite decades of institutional efforts, including mandates from the National Institutes of Health and the FDA, females (particularly females from underrepresented minority groups) continue to be under-enrolled in cardiovascular research [19]. This under-representation has clinical consequences, contributing to reduced efficacy and higher rates of adverse effects in females. These findings underscore the need for systematic approaches to embed gender-sensitive practice into both research and healthcare delivery [11,20]. These patterns may reflect wider systemic and societal issues.

The COVID-19 pandemic and contemporary social movements have significantly influenced discourse on gender inequities in healthcare and medical education. The pandemic threatened progress toward gender equity in academic medicine, as women faculty faced disproportionate challenges from unequal household labour division and may experience reduced academic productivity [21]. Four key social movements (#MeToo, #NiUnaMenos, intersectional feminism, and the global trans rights movement) are transforming health sciences by forcing examination of gendered power relations [22]. The pandemic revealed structural gendered racism as a root cause of health inequities, particularly women of colour who occupy disadvantaged positions in households, occupations, and healthcare institutions [23]. Intersectionality provides a useful conceptual lens, recognising that gender interacts with other axes of identity such as race, class and age, compounding inequities [24,25]. In healthcare, this perspective is vital for understanding how treatment disparities may be shaped by multiple overlapping factors.

Research demonstrates that intersectionality significantly influences healthcare diagnosis, treatment, and outcomes, though this area remains understudied. A systematic review found that while implicit bias research in healthcare exists, studies examining multiple social identity domains (race, sex, class) typically analyse them separately rather than investigating their overlapping effects [26]. In mental health, intersectional analysis reveals that sex, race, class, and ethnicity combine in mutually constitutive ways to explain variation in ADHD diagnosis patterns, demonstrating “paradoxical” stratification effects [27]. Studies confirm that socioeconomic status measures cannot fully account for sex inequalities in health, and that both sex and class affect how risk factors translate into health outcomes through complex intersections [28]. In cardiovascular care, intersectionality approaches are needed to understand how multiple factors interact to influence heart failure patients’ behaviours and health outcomes [29].

Despite recognition of systemic issues, recent empirical evidence on how sex influences actual treatment decisions remains fragmented across specialties, with little synthesis of findings across healthcare systems. A preliminary search of MEDLINE, the Cochrane Database of Systematic Reviews and JBI Evidence Synthesis was conducted and no current or underway systematic reviews or scoping reviews on the topic were identified.

The aim of this scoping review is to synthesise recent evidence (2018–2023) on sex-based differences in the management of patients by healthcare systems, identifying where inequities in treatment provision are most evident.

## 2. Methods

The scoping review was informed by the JBI methodology for scoping reviews [30]. A research question was developed using the PCC (Population, Concept, Context) framework: What recent evidence of measured inequalities in sex and gender are found in the practice of medicine? Key terms were identified, and a search was conducted using Ovid Embase and PubMed databases. MEDLINE (via PubMed) and Embase (via Ovid) were selected as the two principal biomedical databases indexing the clinical literature relevant to the review question [31]. Key terms were adapted for the requirements of each database. The Population was defined as “Patients with any clinical condition or presentation receiving care within healthcare systems.”. The Concept was “Inequalities in the management or treatment of patients based on sex or gender (including differences in diagnosis, access to interventions, procedures, medications, or outcomes).”, with key terms: “sexism”, “gender bias”, “sex bias”, “gender roles”, “gender inequality”, “sex inequality”, “sex factors”, “gender factors”, “gender equity”, “sex equity”, “gender disparities”, “sex disparities”, “gender discordance”, “sex discordance”. The Context was defined as “Healthcare delivery and practice, across specialties and healthcare systems in Europe, the US, Australia, and New Zealand, with key terms: “healthcare”, “medicine”.

The results of both searches were collected, uploaded to and stored in the literature management software EndNote. This scoping review considered both experimental and quasi-experimental study designs including randomized controlled trials, non-randomized controlled trials, before and after studies and interrupted time-series studies. In addition, analytical observational studies including prospective and retrospective cohort studies, case-control studies and analytical cross-sectional studies were considered for inclusion. This review also considered descriptive observational study designs including case series, individual case reports and descriptive cross-sectional studies for inclusion. Qualitative studies were considered if they presented primary data. Grey literature, opinion and editorial articles, reviews and articles without primary data were not considered in this review. Full eligibility criteria were predetermined and used to screen the literature. The inclusion criteria were: written in English, published in or after 2018, published in peer reviewed journals and reporting primary data; address sex/gender differences affecting real patients; compares the healthcare received by patients of different genders. The exclusion criteria were: written in languages other than English, published before 2018, grey literature, opinion and editorial articles, reviews and articles without primary data; address sex/gender differences affecting virtual or simulated patients, healthcare professionals, or in authorship of academic publications; descriptions of differences in presentation between sex/gender without comment on the healthcare they receive, descriptions of the knowledge or attitudes of healthcare practitioners rather than their actions/the actions of systems towards patients, descriptions of patients’ opinions on how healthcare should be apportioned, descriptions about barriers to access or lack of representation, only discussing interventions to previously identified inequalities, reviews the framing of disease in news articles, economic analysis.

Both authors independently screened the literature against the a priori defined eligibility criteria and disagreements were resolved through discussion between the authors. Quality appraisal of the included papers was undertaken using the JBI appraisal checklists [32]. The JBI appraisal checklist is a widely-used method for assessing quality of evidence in healthcare research [33].

A spreadsheet with pre-determined columns was populated with information from each study, including year, authors, country of origin, participants, study type, outcomes measured, results, level of evidence and JBI checklist risk of bias results.

To identify themes and concepts across the included studies, a thematic synthesis approach was used [34]. Quantitative and qualitative data from each study were compared and summarised qualitatively.

## 3. Results

**Fig 1:**
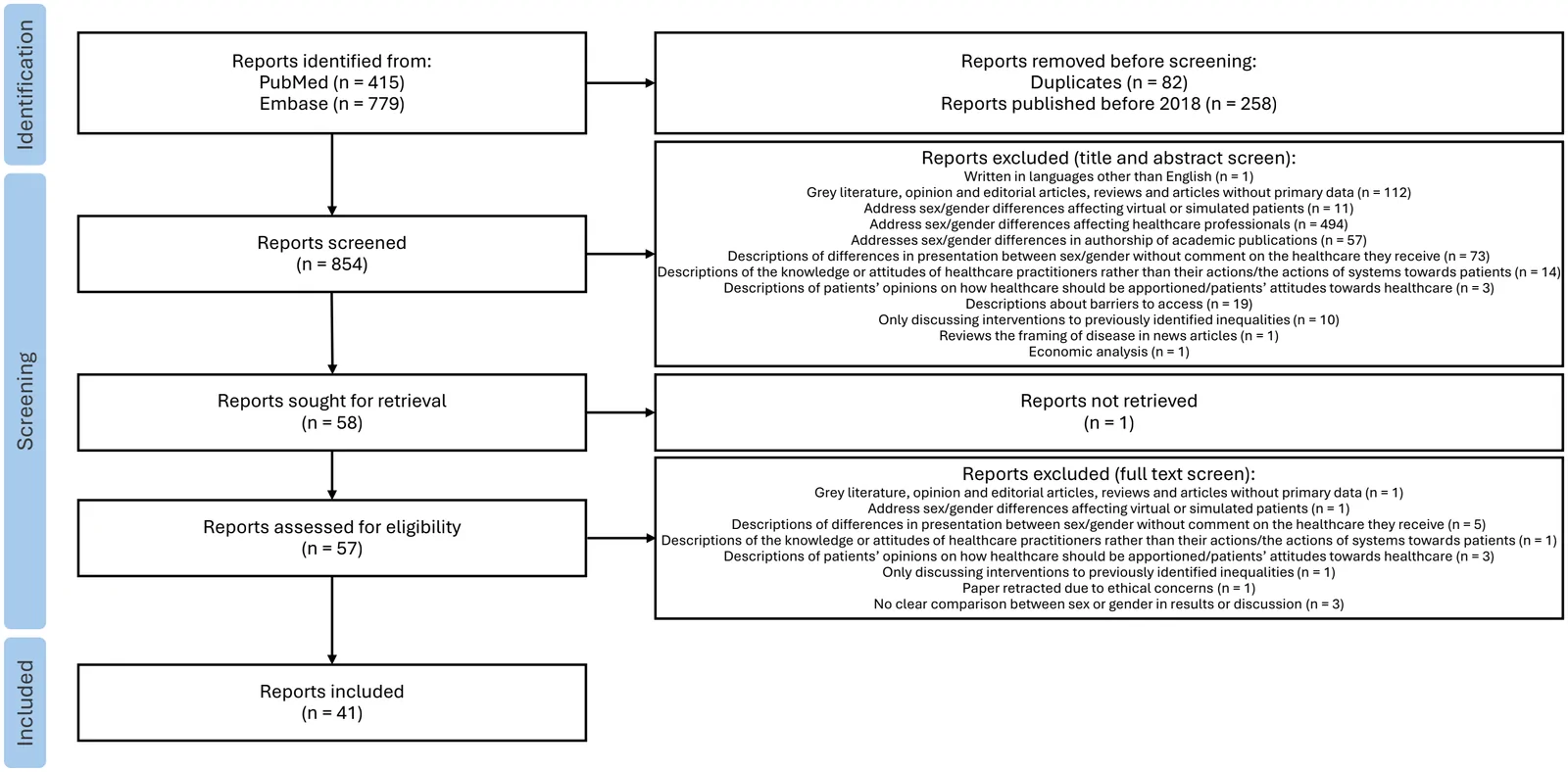
PRISMA flow chart.

The initial search of OVID Embase and PubMed after exclusion of duplicates returned 1,112 results. Title and abstract screening reduced this to fifty-seven results. Full text screening resulted in forty-one papers remaining for analysis (see Fig 1). The most common reason for non-inclusion of papers was that the study focused on differences in the treatment of healthcare professionals by gender rather than the treatment of patients. See Fig 1 for full information on reasons for exclusion.

Of the forty-one papers included in this study, twenty-six were conducted in the United States [35–60], two in the United Kingdom [61,62], one in Canada [63], one in China [64], two in Germany [65,66], one in Portugal [67], one in the Netherlands [68], one in Norway [69], two in Sweden [70,71], one in Romania [72], one in Chile [73], one in Israel [74] and one across Europe [75]. The studies were published in twenty-seven different journals between 2018 and 2023.

Thirty-eight were retrospective case series. One study combined retrospective case series with survey of participants, one combined retrospective case series with prospective case series and survey of participants and one study used mixed methods.

The papers were grouped by specialty area of medicine to aid the development of a synthesised narrative. Fifteen were from cardiovascular medicine [35,36,40–42,49,50,52,54,57,61,65,67,71,72], four from neurology [37,44,60,69], one from renal medicine [38], four from surgical medicine [39,56,63,66], three from transplant medicine [43,59,75], three from emergency medicine [45,51,55], three from endocrinology [46,47,68], three from psychiatry [48,62,70] two from medico-legal [58,64], one from anaesthetics [53], one regarding waiting times across the health system [73] and one from primary care [74].

For the thirty-eight papers that used a retrospective case series, nine did not include multivariate analysis as part of their statistical analysis. Seventeen did not include the ethnicity of the participants. Thirteen did not include comorbid conditions in their description of the participants.

As all included papers were retrospective reviews of medical records, all results are assumed to be relating to the administrative sex of the patients. None of the included papers made any comment about the gender of patients, as opposed to sex. None of the reviews included any patients who were intersex.

Of the thirty-eight retrospective case series, thirty-three noted a statistically significant difference between the treatment of male and female patients. Of the twenty-nine case series using a multivariate analysis, twenty-five studies found a statistically significant difference in treatment between male and female patients.

### 3.1. Descriptive results by specialty

#### 3.1.1. Cardiovascular.

For cardiovascular conditions such as myocardial infarction, heart failure and supraventricular tachycardia, females are more likely to be managed conservatively with medication when compared to invasive procedures such as coronary artery bypass grafting, percutaneous coronary intervention or ablation therapy [41,42,50,61,67]. One study showed that females had worse mortality when presenting with cardiovascular conditions [50], however the others that examined this found no significant difference in mortality [35,61]. In a Swedish study, the time from experiencing the first symptoms of a myocardial infarction to receiving a diagnostic electrocardiogram was longer for females [71]. The discharge destination was examined in one study showing that females were more likely to be discharged to a skilled nursing facility after having a myocardial infarction, likely a marker of worse clinical outcomes [35]. For patients with atherosclerosis of vessels, all studies found females were less likely than males to receive lipid lowering therapies [36,40,54,65]. In patients with diabetes, there was no statistically significant difference between the prescription of lipid-lowering therapies in males and females [72]. During elective cardiovascular surgery females were more likely to receive allogeneic blood transfusion (which has a well-established link with poorer outcomes) [49]. Post-aortic repair surgery, statistically significantly fewer females were discharged with optimal medical management [57].

The group-dynamics of the teams deciding on allocation of advanced heart-failure therapies (AHFT) affected the allocation process. Teams which functioned well were more likely to allocate AHFT to females than males, teams which functioned less well were more likely to allocate AHFT to men than women.

#### 3.1.2. Neurology.

For patients with Parkinson’s disease, men were more likely to be referred for consideration of deep brain stimulation devices, an implantable device to help manage symptoms of Parkinson’s disease. After referral, there was no statistically significant difference in whether males and females went on to have the procedure. For females who did not undergo surgery this was more likely than in males to be due to patient preference [37]. For patients with dementia, females were more likely to receive treatment [44]. For patients admitted to hospital with pneumonia, males were more likely to be screened for underlying neurological causes with a speech and language consult [60]. In the data from a large trial of thrombolysis (an emergency treatment for stroke) no significant difference was found between the treatment of males and females [69].

#### 3.1.3. Endocrinology and diabetes.

For patients with diabetes, studies found no difference in the management between males and females [47,68]. For children, a study found no difference in the number of males vs females who are overweight being screened for diabetes. Males who were obese were more likely than females to be screened for diabetes [46].

#### 3.1.4. Renal and transplant medicine.

For the papers that looked at likelihood of liver transplantation, both found that males were more likely to receive a liver transplant than females [43,75]. Females on the transplant list were more likely to be hospitalised and more likely to be transplanted from the intensive care unit [59].

For dialysis a permanent means of access is preferred to a central venous catheter, females who required dialysis were found to spend longer using a central venous catheter and were less likely to transition to a permanent means of access [38].

#### 3.1.5. Surgery.

Length of stay in hospital (a negative outcome) was longer for females than males in two of three surgical studies [39,66]. There was no difference in discharge destination found for males and females who had elective adult spinal deformity correction [39]. Endovascular treatment is a preferred treatment option for hernia repair operations however females were more likely to receive open hernia repairs [66].

When older patients were studied in Canada, females were less likely to be offered an operation [63]. The outcomes for males and females who had been offered an operation were not significantly different [63]. Prior to surgical management for symptomatic spinal stenosis, females were advised to trial significantly more non-operative treatments [56].

#### 3.1.6. Emergency medicine.

Females were less likely to receive treatment from prehospital emergency medical teams both with targeted temperature management for out of hospital cardiac arrests [45], and opioids for pain management [51]. Bystanders were less likely to use a defibrillator on females in cardiac arrest [55]. A decision to terminate resuscitation efforts was less likely in females in cardiac arrest compared with males. Females were more likely to survive to hospital admission compared with men [55].

#### 3.1.7. Anaesthetics.

Females were less likely to receive an appropriate size of endotracheal tube for their height, receiving on average tubes too large for them [53].

#### 3.1.8. Psychiatry.

Males were less likely to receive a diagnosis of bipolar disorder than females [48]. Females were less likely to be treated with clozapine for treatment resistant schizophrenia [62]. Males with depression are more likely to be prescribed medications [70] but after taking into account six month follow up, are equally likely to be sick-leave certified [70].

#### 3.1.9. Waiting times.

In a comparison of waiting times between males and females in Chile, sixteen conditions were studied [73]. After multivariate analysis, in nine there was no significant difference in waiting times between males and females. For two conditions males waited for significantly longer and in five females waited significantly longer than males [73].

#### 3.1.10. Primary care.

In a review of primary care delivery to geriatric patients in Israel, females were vaccinated at a lower rate [74]. Females were prescribed benzodiazepines at a higher rate than males [74].

#### 3.1.11. Medico-legal.

For drug adverse events that were documented by health-care professionals, more were documented for females. The adverse reactions documented for females were less serious than the events documented for males [58]. One of the most common adverse drug events for females listed was “feeling hot”, however for males one of the most common adverse drug events listed was “death” [58].

A Chinese study of medical errors in elderly patients found numerical differences in the rates of reported errors between males and females between specialties, however there was no statistical analysis of the cases [64]. Females were more likely to report medical errors in nephrology, respiratory, oncology, primary care and internal medicine. Males were more likely to report medical errors in the emergency department, general surgery and medical technology departments. Incidence of errors in the orthopaedic department was the same between males and females [64].

### 3.2. Results of thematic analysis

#### 3.2.1. Interventional procedures.

A theme which emerged during the analysis was papers studying the likelihood of patients of different sex’s having interventional procedures. The ten papers which studied this question are summarised in Table 1 below. The effect direction and estimate refer to the likelihood of female patients receiving an interventional procedure as compared with male patients.

**Table 1:** Likelihood of interventional procedures. AOR = adjusted odds ratio, uOR = unadjusted odds ratio, NR = not recorded, asHR = adjusted subdistribution hazard ratio, uIRR = unadjusted incidence rate ratio.

Study	JBI bias risk	Level of evidence	Effect direction	Effect estimate (95% confidence interval)	p-value
Sacks, N.C. et al. (2020)					
Ablation for paroxysmal supra-ventricular tachycardia	Moderate	Level 4	**▼** (decreased)	aOR 0.83 (0.70–0.98)	0.0278
Verghese, D. et al. (2022)					
Coronary angiography	Moderate	Level 4	**▼** (decreased)	uOR 0.65 (0.64–0.66)	<0.001
Early angiography	Moderate	Level 4	**▼** (decreased)	uOR 0.70 (0.69–0.71)	<0.001
Percutaneous coronary intervention	Moderate	Level 4	**▼** (decreased)	aOR 0.69 (0.68–0.70)	<0.001
Jackson, A. M. et al. (2020)					
Coronary angiography	Low	Level 4	**▼** (decreased)	aOR 0.66 (0.60–0.73)	<0.0001
Percutaneous coronary intervention	Low	Level 4	**▼** (decreased)	aOR 0.60 (0.54–0.66)	<0.0001
Araujo, C. et al. (2018)					
Coronary angiography: STEMI group	Moderate	Level 4	**▼** (decreased)	aOR 0.61 (0.41–0.90)	0.0138
Coronary angiography: NSTEMI group	Moderate	Level 4	— (no effect)	aOR 0.79 (0.62–1.01)	≥ 0.05
Shpiner, D. S. et al. (2019)					
Referral for DBS surgery	Moderate	Level 4	**▼** (decreased)	uOR 0.51 (0.37–0.71)	<0.0001
Proceeding with DBS surgery after referral	Moderate	Level 4	— (no effect)	uOR 0.94 (0.52–1.68)	0.8220
Darden, M. et al. (2021)					
Liver transplant	Moderate	Level 4	**▼** (decreased)	aOR 0.854 (NR)	0.002
Liver ablation	Moderate	Level 4	▲ (increased)	aOR 1.623 (NR)	0.000
Liver resection	Moderate	Level 4	**▼** (decreased)	aOR 0.918 (NR)	0.021
Listabarth, S. et al. (2022)					
Liver transplant	Moderate	Level 4	**▼** (decreased)	aOR 0.78 (0.73–0.82)	<0.0001
Arya, S. et al. (2020)					
Transition to permenent means of access for dialysis	Low	Level 4	**▼** (decreased)	asHR 0.96(0.93–0.98)	<0.001
Rucker, D. et al. (2019)					
Operative management	Low	Level 4	**▼** (decreased)	uOR 0.74 (0.56–0.97)	0.03
Tandon, V. et al. (2020)					
Number of procedures	Moderate	Level 4	**▼** (decreased)	uIRR 0.84 m(NR)	<0.05
Breathett, K. et al. (2023)					
Allocated to advanced therapies	Moderate	Level 4	▲ (increased)	NR	NR

#### 3.2.2. Guideline compliant care/ optimal medical management.

Another theme which emerged during the analysis was papers studying the likelihood of patients of different sex’s having care which was compliant with guidelines. The seventeen papers which studied this question are summarised in Table 2 below. The effect direction and estimate refer to the likelihood of female patients receiving guideline-compliant care as compared with male patients.

**Table 2:** Likelihood of guideline compliant care. aOR = adjusted odds ratio, uOR = unadjusted odds ratio, aHR = adjusted hazard ratio, a% difference = adjusted percentage difference.

Study	JBI bias risk	Level of evidence	Effect direction	Effect estimate (95% confidence interval)	p-value
Shen, X. et al. (2019)					
Statin prescription from cardiologist: commercial insurance plan	Moderate	Level 4	**▼** (decreased)	aOR 0.69 (0.65–0.74)	<0.0001
Statin prescription from cardiologist: Medicare Advantage insurance plan	Moderate	Level 4	**▼** (decreased)	aOR 0.78 (0.76–0.79)	<0.0001
Podell, R. et al. (2018)					
Influenza vaccination	Moderate	Level 4	**▼** (decreased)	uOR 0.83 (0.83–0.84)	<0.0001
Pneumococcal vaccination	Moderate	Level 4	**▼** (decreased)	uOR 0.79 (0.78–0.80)	<0.0001
Mahtta, D. et al. (2020)					
Statin prescription	Moderate	Level 4	**▼** (decreased)	aOR 0.68 (0.62–0.75)	<0.0001
Peters, F. et al. (2020)					
Optimal pharmacological treatment	Moderate	Level 4	**▼** (decreased)	aOR 0.89 (0.86–0.92)	<0.0001
Găman, M. A. et al. (2020)					
Statin prescription	High	Level 4	— (no effect)	uOR 0.62 (0.36–1.08)	0.09
Ilyas, S. et al. (2022)					
Aspirin at discharge	Moderate	Level 4	**▼** (decreased)	uOR 0.74 (0.63–0.86)	0.0001
Statin at discharge	Moderate	Level 4	**▼** (decreased)	uOR 0.66 (0.56–0.76)	<0.0001
Lu, Z. K. et al. (2021)					
Anti-dementia medications	Moderate	Level 4	▲ (increased)	aOR 1.71(1.19–2.45)	0.0036
Stodolski, M. et al. (2020)					
Endoscopic hernia repair	Moderate	Level 4	**▼** (decreased)	uOR 0.29 (0.19–0.46)	<0.0001
Morris, N. et al. (2021)					
Targeted temperature management	Moderate	Level 4	**▼** (decreased)	aOR 0.90 (0.87–0.92)	<0.001
Wimbish, L. et al. (2022)					
Opioid administration	Low	Level 4	**▼** (decreased)	aOR 0.90(NR)	0.004
Wesley, E. et al. (2021)					
Clozapine prescription	Low	Level 4	**▼** (decreased)	aOR 0.66 (0.44–0.97)	<0.05
Ihle-Hansen, H. et al. (2022)					
Onset of symptoms to admission	Moderate	Level 4	— (no effect)	Mean 91.6 vs 95.3 min	0.2900
Door to needle time	Moderate	Level 4	— (no effect)	Mean 38.6 vs 37.0 min	0.3100
Ahmed, F. et al. (2022)					
Prescribed statin therapy	Low	Level 4	— (no effect)	uOR 1.14 (0.61–2.12)	0.682
Received appropriate intensity statin therapy	Low	Level 4	— (no effect)	uOR 1.14 (0.64–2.00)	0.658
Bak, L. et al. (2023)					
19 diabetes process measurements	Moderate	Level 4	— (no effect)	aORs ranged from 0.87–1.06	All >=0.06
Roberts, M. M. et al. (2023)					
Aspirin	Moderate	Level 4	**▼** (decreased)	a% difference −4.36 (−5.38--3.34)	<0.001
Cholesterol management	Moderate	Level 4	**▼** (decreased)	a% difference −3.88 (−4.63--3.14)	<0.001
BP control	Moderate	Level 4	▲ (increased)	a% difference 1.80 (1.28–2.32)	<0.001
Smoking cessation counselling	Moderate	Level 4	▲ (increased)	a% difference 1.67 (0.95–2.38)	<0.001
Bolinger, C. et al. (2020)					
Speech and language consult	Moderate	Level 4	**▼** (decreased)	uOR 0.73 (0.58–0.91)	0.0051
Vajravelu, M. E. et al. (2021)					
Diabetes screening in overweight patients	Moderate	Level 4	▲ (increased)	aHR 1.67 (1.43–2.00)	<0.0001
Diabetes screening in obese patients	Moderate	Level 4	▲ (increased)	aHR 1.43(1.43–1.67)	<0.0001

### 3.3. Analysis of intersectional identities

Out of the forty-one papers, twenty nine did not have result reporting for identities other than sex. Where results were reported for other identities such as age or ethnicity, these were presented separately in seven papers. Five papers gave results for intersectional identities, all of these reported the intersection between sex and ethnicity. See Table 3 below for a summary of these results.

**Table 3:** Intersectional identity results. aOR = adjusted odds ratio, uOR = unadjusted odds ratio, aHR = adjusted hazard ratio, a% difference = adjusted percentage difference, aCE = adjusted estimate of co-efficient, NR = not reported.

Study	JBI bias risk	Level of evidence	Effect direction	Effect estimate (95% confidence interval)	p-value
Darden, M. et al. (2021)					
Caucasian Women transplant	Moderate	Level 4	**▼**(decreased)	0.82 (0.73–0.91) [aOR]	<0.001
African-American women transplant	Moderate	Level 4	**▼**(decreased)	0.42 (0.32–0.54) [aOR]	<0.001
Caucasian women resection	Moderate	Level 4	▲ (increased)	1.597 (1.446–1.765) [aOR]	<0.001
African-American women resection	Moderate	Level 4	▲ (increased)	1.579 (1.303–1.913) [aOR]	<0.001
Caucasion women ablation	Moderate	Level 4	**▼**(decreased)	0.918 (0.849–0.993) [aOR]	0.033
African-American women ablation	Moderate	Level 4	**▼**(decreased)	0.813 (0.684–0.967) [aOR]	0.020
Ahmed, F. et al. (2022)					
White males vs white female statin prescription	Low	Level 4	**—** (No Effect)	0.68 (0.25–1.90) [aOR]	0.4600
Non-white females vs white female statin prescription	Low	Level 4	**—** (No Effect)	Effect)0.36 (0.12–1.06) [aOR]	0.0600
Non white males vs white female statin prescription	Low	Level 4	**—** (No Effect)	2.19 (0.53–8.98) [aOR]	0.2800
Tandon, V. et al. (2020)					
Number of procedures	Moderate	Level 4	Native american women had the lowest average number of procedures, with asian men having the highest	NR	NR
Breathett, K. et al. (2023)					
Race x gender with group function score probability of allocation to advanced heart failure therapies	Moderate	Level 4	**—** (No Effect)	−0.37 (−3.453-2.713) [aCE]	0.815

## 4. Discussion

Gender based differences in the treatment of patients were unable to be studied in this scoping review due to the lack of research found containing information on the gender of patients. This is likely due to the reliance on electronic health records for the papers studied in this scoping review. The electronic health records contain information on the administrative sex of patients, rather than their gender. This issue could be addressed using prospective studies designed specifically to answer questions regarding gender- based inequality in the current healthcare system. The HL7 gender harmony model is a logical model which gives a framework for healthcare organisations to document additional information regarding a patient’s sex and gender [14]. If this model or similar were to be adopted into wide-spread use, research into gender inequality within healthcare could be undertaken at a larger scale.

The results of this scoping review indicate that males and females are frequently treated differently by healthcare systems, though the evidence does not consistently clarify whether such differences confer disadvantage to either sex. Many studies, excluded from the formal part of this review, did not directly measure patient care but instead examined professional attitudes or workforce equity, which fall outside the central focus here. The studies that did examine management revealed variation in treatment practices, raising questions about whether these reflect appropriate clinical considerations or systemic inequities. Notably, very few papers explicitly referenced clinical guidelines recommending sex-specific management, leaving uncertainty about the drivers of observed disparities. Observed differences in treatment practices may relate to patient choice or barriers to access treatment. These findings align with other adjacent reviews, which have synthesised evidence of gender inequities across multiple medical specialties, revealing persistent disparities in both clinical care delivery and professional representation. Grinberg & Sela [76] identified significant gender biases in pain management, with women receiving inadequate analgesia during medical procedures due to unconscious biases, lack of sex-specific protocols, and cultural stereotypes. Shaw et al. [77] examined cardiovascular medicine, emphasizing that achieving equitable care requires consideration of unique gendered structural determinants of health and development of women-optimised care pathways. Ros et al. [78] conducted a broader scoping review revealing substantial inequities in women’s representation across medical professions, particularly in leadership positions and senior faculty roles, with only 13% of identified studies addressing potential interventions. Parsons Leigh et al. [79] focused on critical care medicine, noting it has among the lowest female representation and proposing comprehensive strategies to synthesise and implement solutions for improving gender equity.

Most included studies originated from the United States, limiting the generalisability of findings. This raises the need to examine evidence from other regions such as Europe, the UK, and Australasia, where healthcare systems and policy frameworks differ. Research evidence reveals significant sex inequities in healthcare delivery across the UK and Europe. In the UK’s National Health Service, females and older people receive guideline-recommended cardiovascular treatments less frequently than males and younger people, with limited research available on ethnic disparities [80]. A broader European analysis found that after adjusting for healthcare needs, significant pro-rich inequity emerged in half of studied countries for physician contacts, primarily due to higher-income groups’ greater use of specialist services while lower-income groups relied more on general practitioner care [81]. Sex bias in healthcare manifests in two ways: assuming similarities between male’s and female’s health situations when differences exist, and assuming differences where similarities occur, both potentially leading to negative health outcomes for females [82]. Despite equity being a central NHS objective since 1948, important inequities persist in UK healthcare access, though methodological limitations often prevent firm conclusions about underlying causes [83]. This regional evidence reinforces the broader pattern identified in this review, showing that inequities in healthcare delivery are not confined to the US context but also persist across European systems.

A consistent trend was observed: females were statistically less likely than males with similar clinical characteristics to be offered invasive management, and when managed conservatively, females were statistically less likely to receive optimal pharmacological therapy. It is not possible to determine whether this was as a result of inequities or due to another cause. Breathett et al. studied how group dynamics may influence the allocation of treatment to patients and found that worse group dynamics resulted in females and patients of colour being less likely to be allocated intensive treatment options.

Much of the recent research on sex differences in management has been conducted in cardiovascular medicine, reflecting the historical prominence of this field since early studies on sex-based disparities [82]. It remains unclear whether this concentration reflects a true predominance of inequities in cardiovascular care or simply research inertia building on prior work.

Research has identified several explanations for observed sex differences in healthcare delivery. Raine [84] found evidence of sex disparities across multiple conditions, with males more likely to receive certain procedures like renal transplantation and HIV treatments, while females were more likely to undergo liver transplantation and cataract surgery. The study attributed these differences to both demand factors (disease prevalence, severity, patient preferences) and supply factors (clinical judgment). With patient preference also being attributed to the reduced implantation of deep-brain stimulation devices in females in one study in this review [37]. Elderkin-Thompson & Waitzkin [85] highlighted communication differences as a key factor, noting that physicians commonly attribute women’s symptoms to “over anxiousness” and make more diagnostic errors with female patients, even when positive test results are present. These explanations provide possible mechanisms underlying the consistent trend observed in this review, where females are statistically less likely than males to receive invasive management or optimal pharmacological therapy.

Although thirteen studies reported results comparing outcomes for groups other than males and females, only four presented the results using intersectional identities. The results of these studies are summarised in Table 3, no clear conclusions could be drawn due to the lack of data. The reporting of results for different identities separately rather than in an intersectional manner has been noted in previous reviews [26].

The evidence base remains limited in quality. Many studies relied on retrospective analyses of medical records, with incomplete data limiting the strength of conclusions. Several did not perform multivariate analyses to adjust for potential confounders such as race, comorbidity, or socioeconomic status. Future research should prioritise prospective designs to establish whether differences are attributable to sex itself rather than intersecting factors.

Several major international funding agencies have implemented policies requiring or encouraging sex and gender analysis in health research. The National Institutes of Health (NIH) has instituted policies requiring researchers to include appropriate populations and analyse data accordingly, focusing on sex as a biological variable (SABV) to enhance reproducibility [86,87]. The Canadian Institutes of Health Research (CIHR) implemented a sex- and gender-based analysis (SGBA) policy, with mandatory questions introduced in 2010 showing increased compliance over time, though disparities across disciplines persist [87,88]. The European Commission requires integration of the “gender dimension,” incorporating sex, gender, and intersectional analysis into research and innovation [87]. A comprehensive analysis of 22 major national funding agencies across six continents reveals varying approaches to implementing sex, gender, and diversity analysis policies, highlighting opportunities for improved international collaboration and research excellence [89].

A limitation of this paper relates to the methodology employed. Scoping reviews in health research face several common methodological limitations that must be acknowledged when interpreting findings. Key limitations include a lack of prospective studies, non-representative samples that limit generalizability, and insufficient data on mediators and moderators of relationships between variables [90]. The heterogeneous nature of literature in scoping reviews creates challenges in gathering, analysing, and interpreting information, with epistemological considerations significantly influencing methodological decisions [91]. The search was restricted to two biomedical databases and did not include allied health or multidisciplinary indexes such as CINAHL, so relevant studies indexed solely in those sources may have been missed. Consistent with the mapping aim of a scoping review, the consistency of effect directions observed across specialties suggests the principal patterns are unlikely to be materially altered by a small number of additional records.

## 5. Conclusions

This review demonstrates that recent evidence points to persistent sex-based differences in the treatment of patients. Across multiple specialties, the most consistent finding was that females were statistically less likely than males with comparable clinical characteristics to receive invasive or intensive treatments, and when treated conservatively, were less likely to receive guideline compliant therapy. Important gaps in evidence quality exist, with many studies limited by retrospective designs, incomplete adjustment for confounders, and a predominance of US-based data. A gap in research investigating the gender of patients rather than administrative sex was evident. Future research must integrate equity considerations and intersectional analysis into study design, employ rigorous analytical methods, and extend to diverse healthcare systems. Addressing these gaps will be critical to determining whether observed differences represent appropriate clinical variation or systemic inequities, and to ensuring that practice reflects equitable standards of care.

## Supporting information

S1 File Appendix 1. (DOCX)

S2 File Supplementary material. (XLSX)
