# Supplementary material for: Measured treatment differences between sexes and genders: A scoping review
Source: PLoS One. 2026 Sep 16;21(9):e0356176. doi: 10.1371/journal.pone.0356176 (PMC13580986; doi:10.1371/journal.pone.0356176)
Supplement: Supplementary file 1 [file pone.0356176.s001.docx]

# Appendix 1

PubMed Search term 01/01/2018 – 19/12/2023

(sexism [tiab] OR "gender bias" [tiab] OR "sex bias" [tiab] OR "gender roles" [tiab] OR "gender

inequality" [tiab] OR "sex inequality" [tiab] OR "sex factors" [tiab] OR "gender factors" [tiab]

"gender equity" [tiab] OR "sex equity" [tiab] OR "gender disparities" [tiab] OR "sex disparities"

[tiab] OR "gender discordance" [tiab] OR "sex discordance"[tiab]) AND ("healthcare" [tiab] OR "medicine" [tiab])

Embase Search term 01/01/2018 – 19/12/2023

('sexism':ti,ab OR 'gender bias':ti,ab OR 'sex bias':ti,ab OR 'gender roles':ti,ab OR 'gender inequality':ti,ab OR 'sex inequality':ti,ab OR 'sex factors':ti,ab OR 'gender factors':ti,ab OR 'gender equity':ti,ab OR 'sex equity':ti,ab OR 'gender disparities':ti,ab OR 'sex disparities':ti,ab OR 'gender discordance':ti,ab OR 'sex discordance':ti,ab) AND ('healthcare':ti,ab OR 'medicine':ti,ab)
